# Supplementary material for: Dynamic pathways in energy landscapes guiding supramolecular Janus dendrimer self-assemblies between lamellar and cubic architectures
Source: Nat Commun. 2025 Aug 29;16:8075. doi: 10.1038/s41467-025-62866-9 (PMC12397278; doi:10.1038/s41467-025-62866-9)
Supplement: Supplementary file 1 — Supplementary Information [file 41467_2025_62866_MOESM1_ESM.pdf]

Supplementary Information

Dynamic pathways in energy landscapes guiding supramolecular Janus dendrimer self-assemblies between lamellar and cubic architectures

Jiabin Luan[1], Danni Wang[1], Niels P. Kok[1], Neshat Moslehi[2], Ilja K. Voets[2], and Daniela A. Wilson[1]*

[1] Institute for Molecules and Materials, Radboud University Nijmegen, Heyendaalseweg 135, 6525 AJ, Nijmegen, The Netherlands

[2] Laboratory of Self-Organizing Soft Matter, Department of Chemical Engineering and Chemistry and Institute of Complex Molecular Systems, Eindhoven University of Technology, P.O. Box 513, 5600 MB, Eindhoven, The Netherlands

*Corresponding author. e-mail: d.wilson@science.ru.nl

Table of Contents

S1 | Experimental: synthesis 3

S1.1 | (3,5)12G1-PE-(3,4)-3EO-G1-$(OCH_3)_4$ 3

S1.2 | (3,5)12G1-PE-(3,4)-3EO-G1-$(OH)_4$ 5

S2 | Experimental: characterization 6

S2.1 | NMR Spectroscopy 6

S2.2 | Mass Spectrometry 6

S2.3 | Light Scattering (LS) 6

S2.4 | Nanoparticles tracking analysis (NTA) 7

S2.5 | Small Angle X-ray Scattering (SAXS) 7

S3 | Supplementary Figures & Discussion 9

S4 | References 31

S1 | Experimental: synthesis

S1.1 | (3,5)12G1-PE-(3,4)-3EO-G1-(OCH$_3$)$_4$

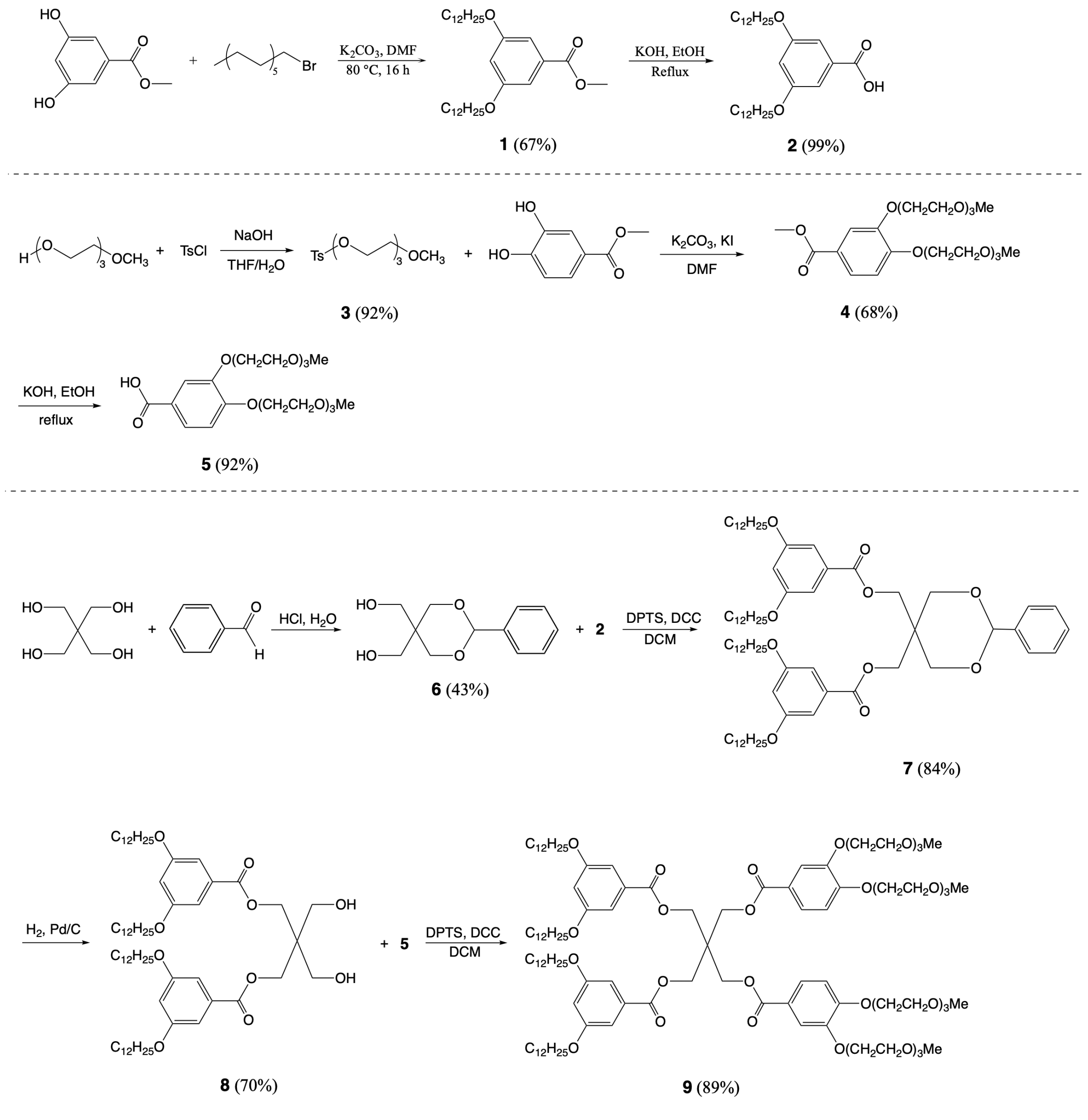

Supplementary Fig. 1 | Synthesis of (3,5)12G1-PE-(3,4)-3EO-G1-(OCH$_3$)$_4$ Janus dendrimer.

The amphiphilic Janus dendrimer (3,5)12G1-PE-(3,4)-3EO-G1-(OCH$_3$)$_4$, consisting of hydrophobic alkyl chains, hydrophilic oligo(ethylene glycol), and a pentaerythritol core, was synthesized through a nine-step procedure, as previously reported.[1]

Synthesis of the hydrophobic unit

The hydrophobic unit was synthesized using a convergent strategy, involving the etherification of 3,5-hydroxybenzoates with 1-bromododecane, followed by hydrolysis of the methyl ester group. This method ensured high yield and selectivity for the desired hydrophobic segment.

Synthesis of the hydrophilic unit

The synthesis of the hydrophilic unit began with the tosylation of methyl-terminated triethylene glycol, followed by its reaction with methyl ester-protected gallic acid via Williamson ether synthesis. Subsequent hydrolysis of the methyl ester group yielded the corresponding acid, completing the hydrophilic segment preparation.

Functionalization of the pentaerythritol core

To functionalize the pentaerythritol core asymmetrically, a benzylidene-protection strategy was employed. This approach facilitated the sequential conjugation of the hydrophobic unit, followed by the hydrophilic unit. The differential substitution ensured precise structural control of the Janus dendrimer.

Purification and characterization

All intermediate products and the final dendrimer were purified using standard chromatographic techniques. Structural confirmation and purity were verified through nuclear magnetic resonance (NMR) spectroscopy and matrix-assisted laser desorption/ionization time-of-flight (MALDI-TOF) mass spectrometry.

This optimized synthesis route provided the desired amphiphilic Janus dendrimer with high structural fidelity and reproducibility, offering a robust platform for further investigations.

S1.2 | (3,5)12G1-PE-(3,4)-3EO-G1-$(OH)_4$

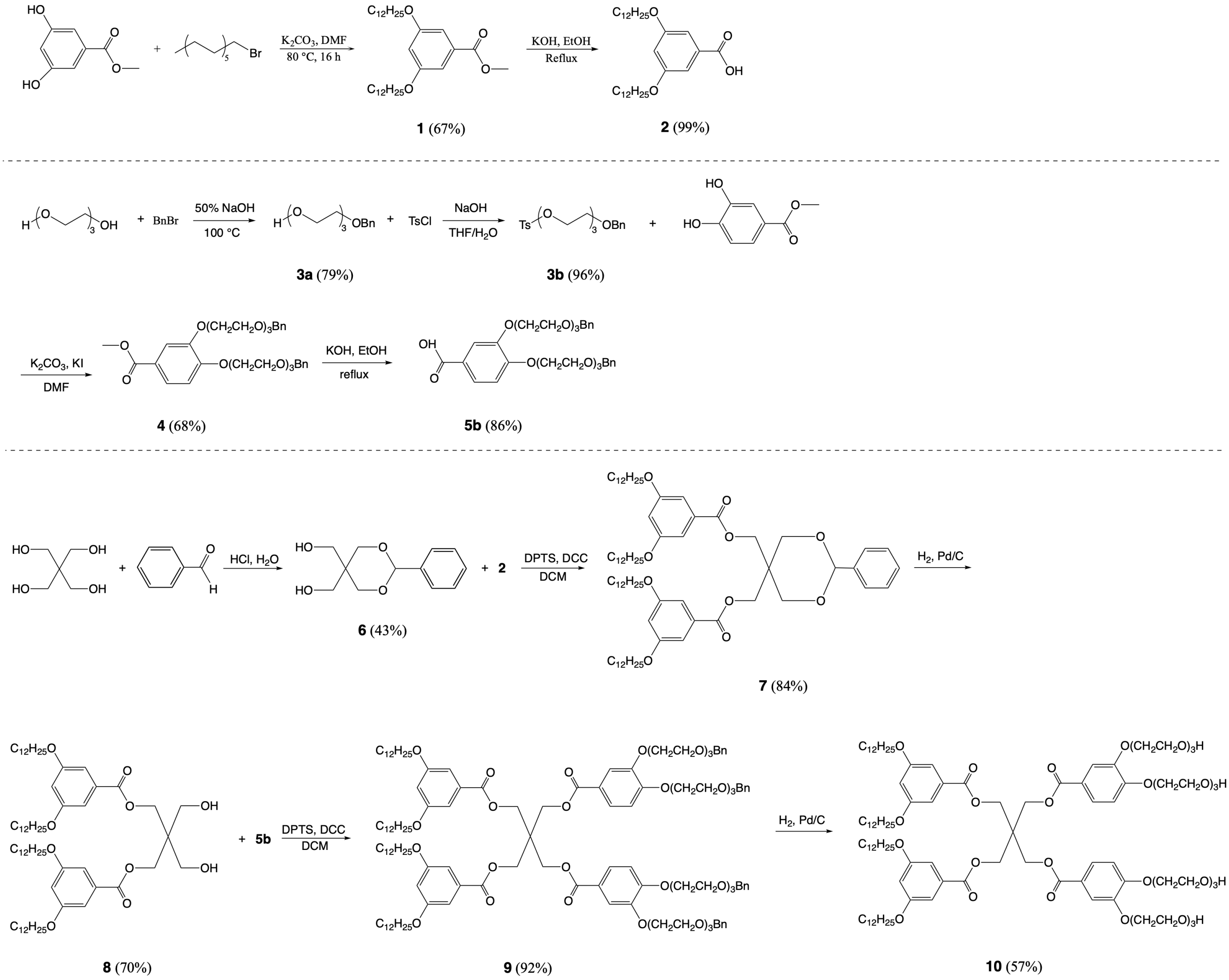

Supplementary Fig. 2 | Synthesis of (3,5)12G1-PE-(3,4)-3EO-G1-$(OH)_4$ Janus dendrimer.

The synthesis of (3,5)12G1-PE-(3,4)-3EO-G1-$(OH)_4$ followed a similar procedure to that of (3,5)12G1-PE-(3,4)-3EO-G1-$(OCH_3)_4$, with a key modification involving the protection of tosylated triethylene glycol using benzyl ether (Bn) (Compound **3a**, **Supplementary Fig. 2**). The hydrophilic chains were subsequently incorporated into the hydrophilic dendrons via Williamson ether synthesis. Orthogonal esterification of the pentaerythritol core was performed to achieve the Janus dendrimer. In the final step, the benzyl protecting groups were removed from the hydrophilic dendrons, yielding hydroxy-terminated end-groups.

All intermediate and final products were meticulously purified and characterized using NMR and MALDI-TOF.

S2 | Experimental: characterization

S2.1 | NMR Spectroscopy

Nuclear magnetic resonance (NMR) spectra were obtained using a Bruker 400 MHz Avance III HD Nanobay spectrometer equipped with a BBFO probe. Tetramethylsilane (TMS, $\delta = 0.0$ ppm) or residual solvent protons in the deuterated solvent were used as internal references for chemical shift calibration. ^{1}H NMR spectra were recorded with 32 or 64 scans and a relaxation delay of 5 seconds to ensure optimal signal acquisition.

S2.2 | Mass Spectrometry

Matrix-assisted laser desorption/ionization time-of-flight (MALDI-TOF) mass spectrometry was conducted using a Bruker Microflex LRF MALDI-TOF system equipped with a 337 µm nitrogen laser and operated in reflection mode. The saturated matrix solution was prepared by dissolving excess α-cyano-4-hydroxycinnamic acid in a 30:70 (v/v) acetonitrile (ACN)/water solution containing 0.1% trifluoroacetic acid (TFA). Analytical samples were prepared by dissolving the analyte in a 1:1 (v/v) ACN/water or tetrahydrofuran (THF) solution at a concentration of 2 mg/mL. Equal volumes of the analyte solution and the matrix solution were mixed thoroughly, and 1.0 µL of the resulting mixture was applied to the MALDI plate. The sample was air-dried at room temperature before being introduced into the vacuum chamber of the instrument. Laser intensity, steps, and applied voltages were optimized as needed based on the specific characteristics of each analyte.

S2.3 | Light Scattering (LS)

Light scattering (LS) measurements were performed using a Malvern Zetasizer Nano-ZS (Malvern Instruments) equipped with a He-Ne laser (633 nm, 4 mW) and an avalanche photodiode detector positioned at a backscatter angle of 173°. These measurements were employed to determine the average hydrodynamic diameter (D_h), polydispersity index (PDI), and derived count rates of the self-assembled structures. For temperature-dependent studies during heating and cooling cycles, samples were equilibrated for 300 seconds at each temperature prior to measurement. Particle size and size distribution were calculated using the Stokes-Einstein equation, based on the analysis of fluctuations in scattered light intensity caused by the Brownian motion of particles.

S2.4 | Nanoparticles tracking analysis (NTA)

Nanoparticle tracking analysis (NTA) was conducted using a Nanosight LM10-HS instrument equipped with a Marlin camera and a 60 mW blue laser (405 nm). NTA enables high-resolution, particle-by-particle analysis, providing detailed information on particle size and concentration. For the analysis, the sample solution (0.5 mg/mL) was diluted 1000 times to achieve an optimized particle concentration of 10^7 to 10^9 particles/mL. The diluted solution was injected into the sample chamber, where the Brownian motion of the nanoparticles was recorded in three separate 60-second videos at a frame rate of 30 frames per second. The particle size and concentration were determined by averaging the results obtained from these video recordings. Multiple measurements were conducted for each condition by loading multiple individual samples for analysis, ensuring the reliability and reproducibility of the data.

S2.5 | Small Angle X-ray Scattering (SAXS)

SAXS measurements were conducted in transmission mode on an in-house instrument, SAXSLAB GANESHA 300XL, equipped with a high brilliance Microfocus Cu Source, Xenocs Genix3D, with wavelength λ = 1.54184 Å. The samples as well as the solvent, Milli-Q water, were loaded into lockable thin wall quartz capillaries with an inner diameter of 2 mm. The scattered X rays were collected on a Pilatus 300 K solid-state photon-counting 2D detector at a q range of 0.013 – 0.670 $Å^{-1}$ with an exposure time of 3 hours. The scattering vector, q, is related to the scattering angle by the following equation:

$$q = {}^{4\pi}/_{\lambda} \sin\theta$$

where λ and 2θ are the wavelength of the incident radiation and the scattering angle, respectively. The two-dimensional images obtained were averaged using SAXSGUI software to obtain the intensity $I(q)$ vs. q profiles. Prior to the measurements, silver behenate was used for q-calibration. The scattering profile of Milli-Q water was used for data subtraction. The subtracted SAXS profiles are displayed as the average intensity $I(q)$ vs. q.

Using the SasView program, we selected a plug-in form factor model composed of a combination of two pre-existing models; i) the *Power-law,* a shape-independent model providing background scattering, and ii) the *Lamellar_hg_stack_caille*[2], which describes the form factor of lamellar structures with separate scattering contributions from the head and tail. In this combined model, the scattering intensity $I(q)$ is:

$$I(q) = 2\pi \frac{P(q)S(q)}{q^2\delta}$$

where δ is the lamellae thickness, and $P(q)$ and $S(q)$ are the form and structure factors, respectively, given by:

$$P(q) = \frac{4}{q^2}\{\Delta\rho_H[sin\,[q(\delta_H + \delta_T)] - sin\,(q\delta_T)] + \Delta\rho_T sin\,(q\delta_T)\}^2$$

$$S(q) = 1 + 2\sum_{1}^{N-1}\left(1 - \frac{n}{N}\right)\cos\,(qdn)\exp\left(-\frac{2q^2 d_{spacing}^2 \alpha(n)}{2}\right)$$

$\alpha(n)$ is a correlation function for the lamellae:

$$\alpha(n) = \frac{\eta_{cp}}{4\pi^2}(\ln\,(\pi n) + \gamma_E)$$

in which the Euler's constant $\gamma_E = 0.5772156649$ and the Caille constant η_{cp} is given by:

$$\eta_{cp} = \frac{(q^*)^2 k_B T}{8\pi\sqrt{K\bar{B}}}$$

where q^* is the position of the first Bragg peak, k_B is the Boltzmann constant, T is the temperature, δ_H and δ_T represent thicknesses of head and tail respectively, N is the number of lamellae, K is the bending elasticity, and $\bar{B}$ is the compression modulus. Finally, $\Delta\rho_H$ and $\Delta\rho_T$ are the differences in scattering length density of the head (compared to the solvent) and tail (compared to the head).

S3 | Supplementary Figures & Discussion

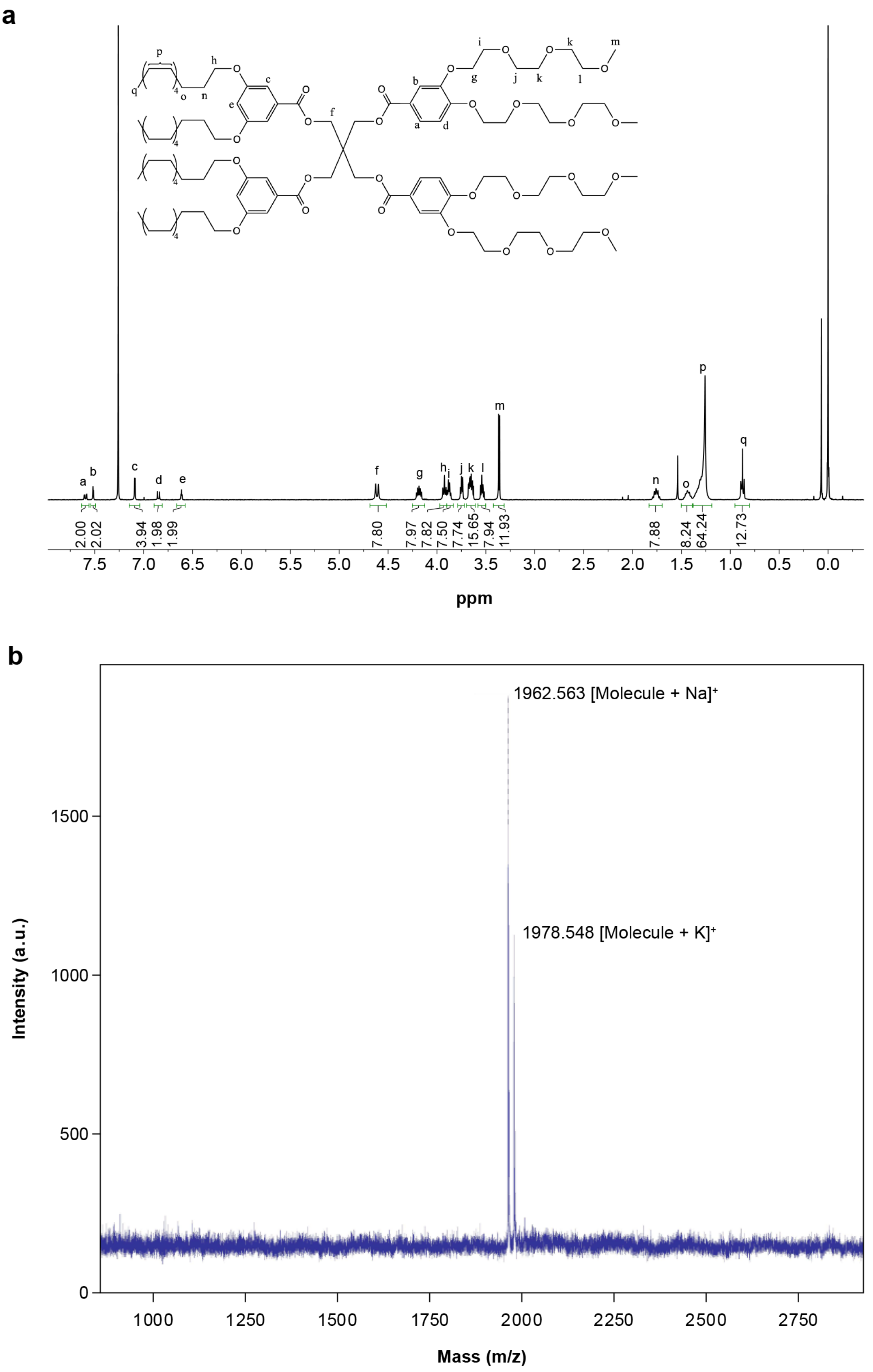

Supplementary Fig. 3 | Characterization of (3,5)12G1-PE-(3,4)-3EO-G1-$(OCH_3)_4$ Janus

dendrimer. a ^{1}H NMR in $CDCl_3$. **b** MALDI-TOF spectra.

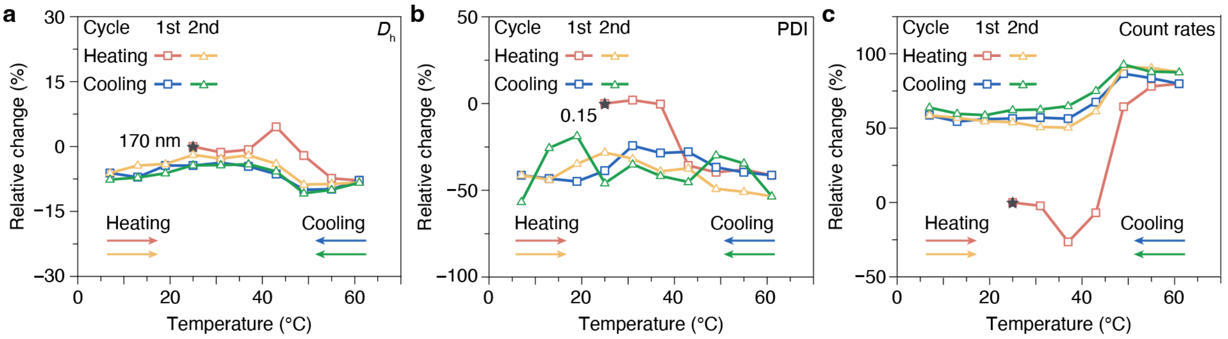

Supplementary Fig. 4 | LS characterization of self-assemblies in the absence of ethanol. Relative changes of (**a**) D_h (%), (**b**) PDI (%), and (**c**) derived count rates (%) of assemblies to the starting points (indicated by the symbol of pentagram) during two heating/cooling cycles in the LS temperature trend measurements.

Supplementary Fig. 4 shows the relative change of D_h, PDI, and derived count rates of assemblies to the starting points (indicated by the symbol of pentagram) during the heating and cooling cycles. In the first heating cycle, we can identify 37 °C, which was characterized by a slight increase in size and a significant decrease in PDI. 50 °C was also noticed in the first heating cycle, which was characterized by a slight decrease in size and an increase in count rates. These changes suggest a potential transition in the states of the assemblies, which was further investigated by LS, NTA, and cryo-TEM in the kinetic studies (**Fig. 2** and **Fig. 3**).

It is noteworthy mentioning that the investigation at body temperature of 37 °C brought insights and attention to the different energy states of self-assemblies of Janus dendrimers, which have been shown great potential in biomedical applications such as antibacterial nanoreactor[3] or targeted mRNA delivery[4-6].

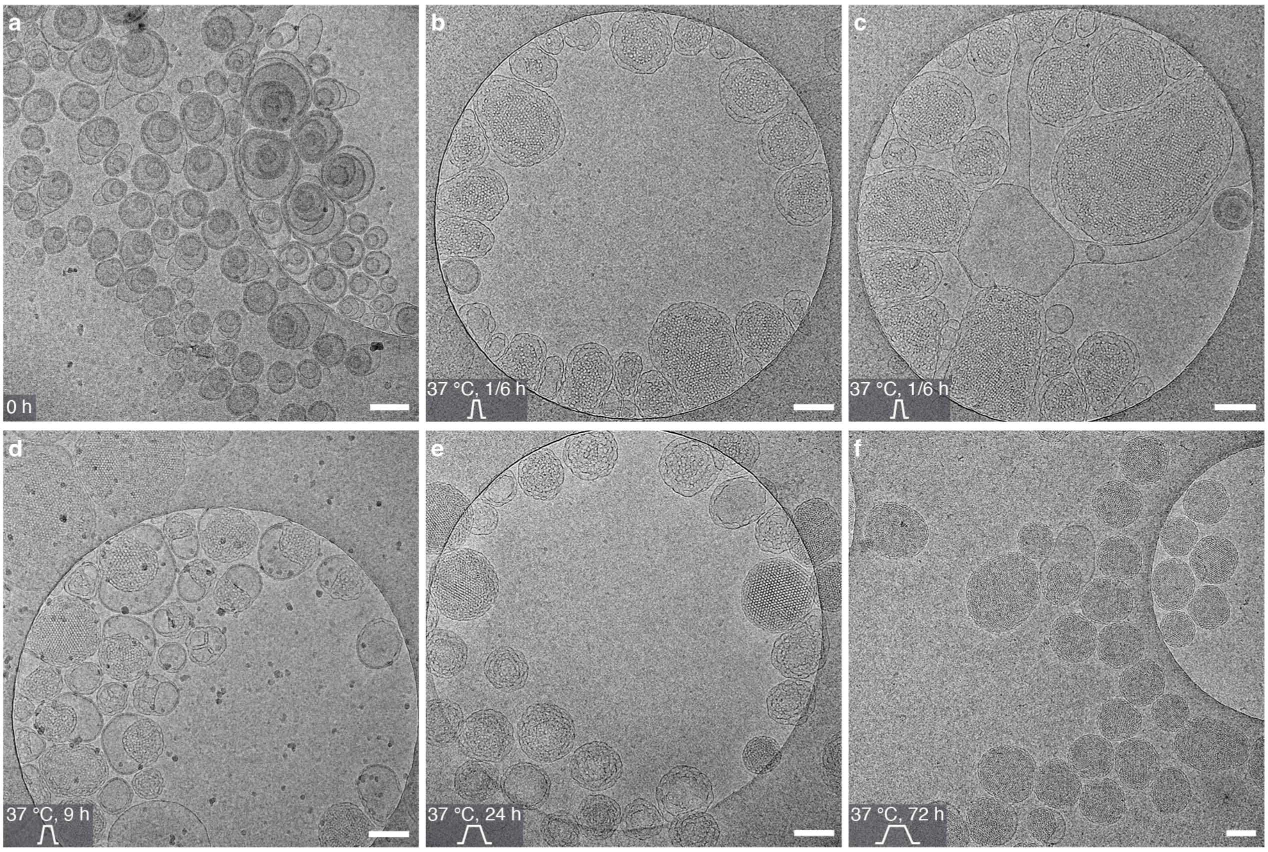

Supplementary Fig. 5 | Kinetic morphological investigation of self-assembled Janus dendrimers via 37 °C annealing in the absence of ethanol. a–f Cryo-TEM images of self-assemblies following ethanol removal (**a**) and assemblies annealed at 37 °C for 10 min (**b,c**), 9 h (**d**), 24 h (**e**), and 72 h (**f**). Scale bars are 200 nm.

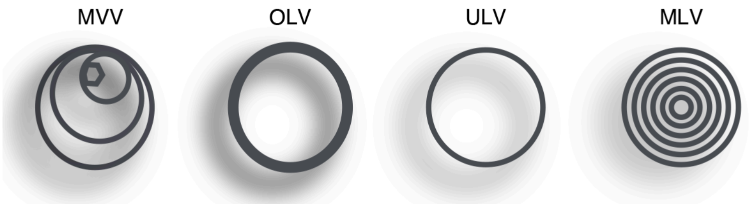

Supplementary Fig. 6 | Vesicle types involved in the current study: multivesicular vesicle (MVV) with nonconcentrically arranged internal vesicles, oligolamellar vesicle (OLV) with a few concentrically arranged internal vesicles, unilamellar vesicle (ULV) with a single lamellae (bilayer), and multilamellar vesicle (MLV) with many concentrically arranged internal vesicles, also known as onion vesicle.

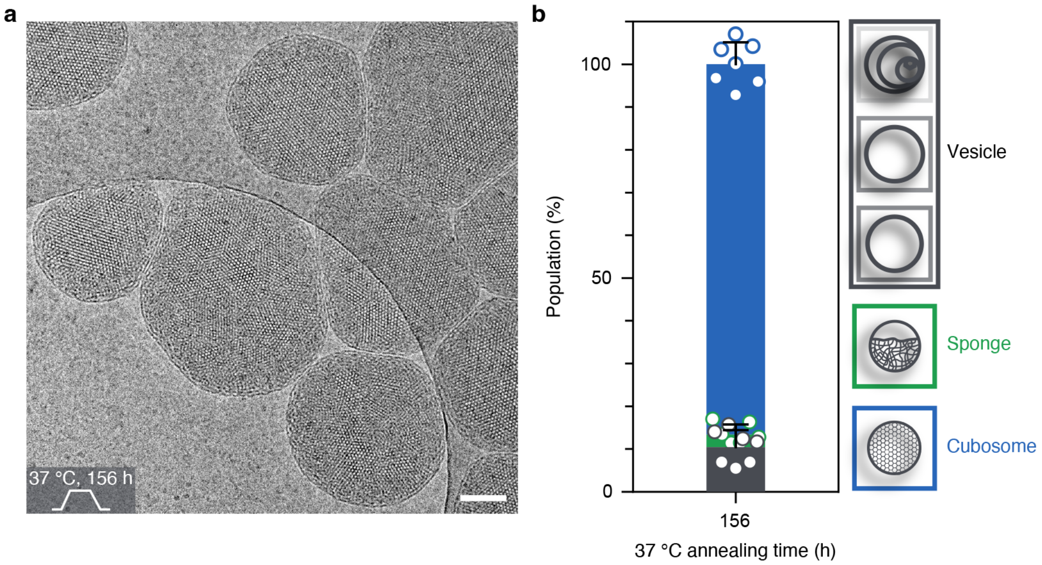

Supplementary Fig. 7 | Stability of the cubosomes at extended 37 °C annealing time. a Cryo-TEM images of self-assemblies annealed at 37 °C for 156 h. Scale bar is 200 nm. **b** Quantitative measurement of assemblies obtained from post-annealing at 37 °C. Images from different areas were taken and counted to minimize the error (n > 500 particles). The bar plot represents mean ± standard deviation (s.d.).

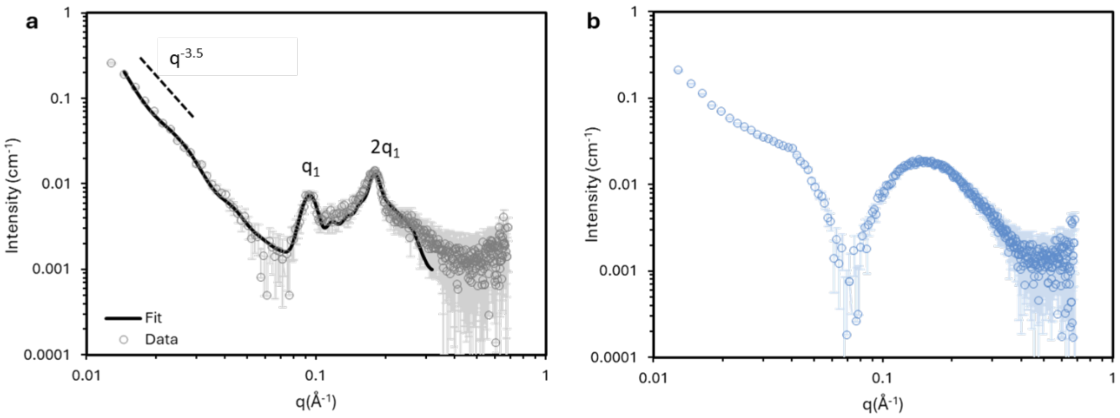

Supplementary Fig. 8 | Structural investigation by SAXS. SAXS profiles of (**a**) the original self-assembly (MVVs) and (**b**) the self-assembly annealed at 50 °C for 10 min (cubosomes) in the absence of ethanol. Data are presented as mean ± s.d.

The small-angle X-ray scattering (SAXS) profiles acquired directly after sample preparation (**Supplementary Fig. 8a**; corresponding cryo-TEM in **Fig. 2d**) and after 10 min annealing at 50 °C (**Supplementary Fig. 8b**; corresponding cryo-TEM in **Fig. 3e**) exhibit clear differences. Neither profile commences with a q-independent plateau at low q-values, indicating that the nanostructures exceed accessible length scale of the SAXS instrument ($R > \pi/q_{\min} \sim 24$ nm). The SAXS curve of the as-prepared sample (**Supplementary Fig. 8a**) shows characteristics typical of vesicular structures, such as power-law scattering $I \propto q^{-3.5}$ in the low-q regime due to the large vesicle size, and two pronounced, equidistant Bragg reflections $q_1 \sim 0.095$ Å^{-1} and $2q_1 \sim 0.183$ Å^{-1}, indicative of a multi-lamellar nature of the MVVs. From the position of the first Bragg reflection q_1, we estimate a lamellar spacing of $d = 2\pi/q_1 = 66$ Å. Upon annealing, the SAXS profile (**Supplementary Fig. 8b**) no longer features the $I \propto q^{-3.5}$ regime or the equidistant Bragg reflections, indicating a substantial change in both morphology and internal ordering.

Having established that the as-prepared and annealed states are distinct in morphology and ordering, we attempted to describe their profiles in more depth using the SasView software to extract structural information using appropriate form and structure factors for MVVs and cubosomes, which were the dominant structures in the corresponding cryo-TEM images (**Fig. 2d** and **Fig. 3e,** respectively). For the as-prepared sample, we selected a modified Caillé model[7-8] to describe the reflections, combined with a power-law function to describe the initial decay in the low-q regime. The solid line in **Supplementary Fig. 8a** represents the fit with this

model to the experimental data (symbols). Fitting Caillé model[2] (see Methods section for parametrization) yielded structural parameters of the lamellar structure. We obtained an average of $N \sim 5$ lamellae, thickness of the dendrimer head region of $\delta_H \sim 4$ Å, thickness of the dendrimer tail region of $\delta_T \sim 12$ Å, and a lamellar spacing of $d = 69.8$ Å (**Supplementary Table 1**). The values of α (power-law) and N were in agreement with a previously reported prediction proposed for the relation between N and α for (non-) concentric MLVs[8], with non-concentric MLVs corresponding to MVVs. It should be mentioned in this context that the number of bilayers (N) for MLV/MVV is related to the compactness of the particles evident from the exponent of the SAXS profile for a smooth surface in the accessible q range. This relation lies between q^{-2} (for hollow spherical lamellar structures with a smooth surface) and q^{-4} (for compact volumes -not hollow- spherical particle, with uniform electron contrast with a smooth surface)[8]. The extracted structural parameters from fitting are provided in **Supplementary Table 1**.

Supplementary Table 1 | Structural parameters extracted from the fit to the experimental SAXS data shown in Supplementary Fig. 8a.

Parameter	α	N	η_{cp}	$d_{spacing}$ (Å)	δ_H(Å)	δ_T(Å)	$\Delta\rho_H$(10^{-6}/Å^2)	$\Delta\rho_T$(10^{-6}/Å^2)
Value	3.5	5	0.18	69.81	3.96	12.11	14.23	-3.08

It is worth noting that discrepancies between bilayer thicknesses derived from SAXS and cryo-TEM measurements have been observed previously. At first glance, this may appear surprising, as both techniques rely on contrast generated by differences in electron density between the bilayer and surrounding solvent. However, these discrepancies have been attributed to fundamental differences in X-ray/matter and electron/matter interactions[9-10], as well as in the methodologies used for data analysis. SAXS data are typically analyzed with a modeling procedure in reciprocal space (through a one-dimensional Fourier transform), whereas cryo-TEM analysis is conducted in real space, based on two-dimensional projections of the electron density of the sample. Importantly, such projections can be affected by the contrast transfer function, as well as imaging parameters such as electron dose and defocus conditions[11]. Therefore, disparities in bilayer thickness estimates between the two techniques are to be expected, and for this reason, we refrain from making a direct quantitative comparison.

Regarding the annealed sample, the identification of the specific type of the bicontinuous cubic phase (e.g., primitive, gyroid, or double diamond) formed after 10 min annealing at 50 °C is not possible based on the available SAXS data, due to the absence of higher-order Bragg reflections[12-13]. This limitation is likely a consequence of restricted long-range order and significant variability in lattice parameters both within individual particles (intraparticle dispersity) and across the sample population (interparticle dispersity), due to the dynamic nature of the cubosomes. Similar challenges have been reported for other complex soft matter systems, where the resolution of SAXS was found to be insufficient for detailed structural assignment[14]. As a result, specific structural features, such as the dimensions of the aqueous channels, could not be determined from the present data.

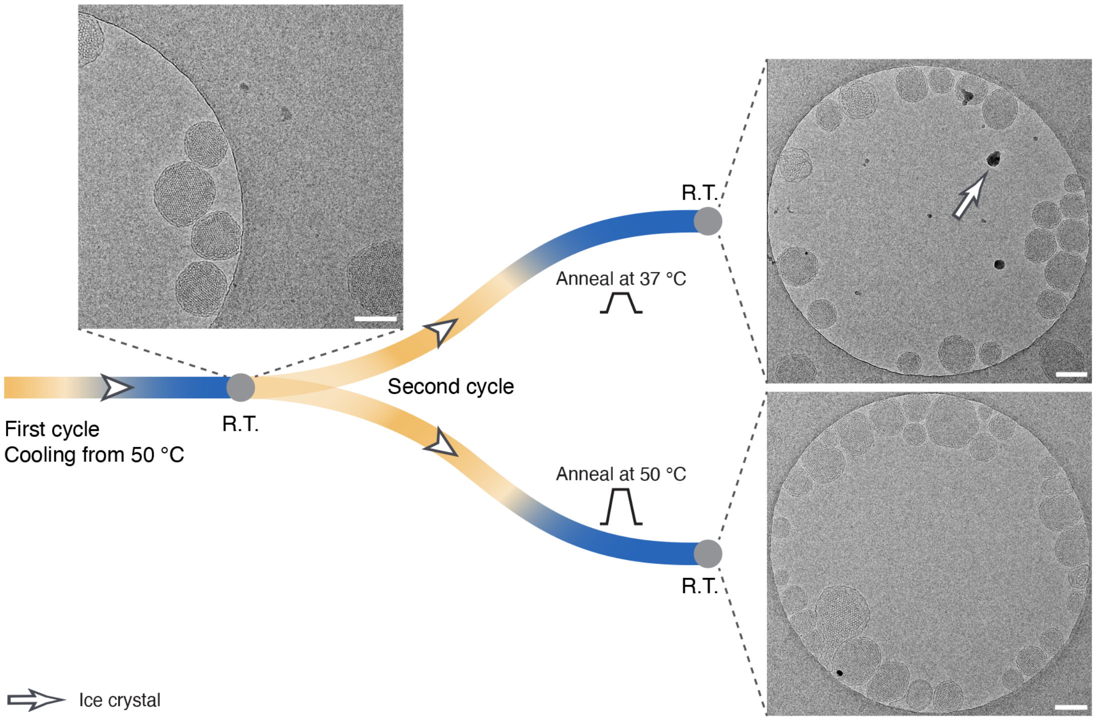

Supplementary Fig. 9 | Investigation of self-assemblies during the second heating/cooling cycle following the initial treatment. Cryo-TEM images depict the self-assemblies at room temperature (R.T.) after annealing at the indicated temperatures during the second cycle. Scale bars are 200 nm.

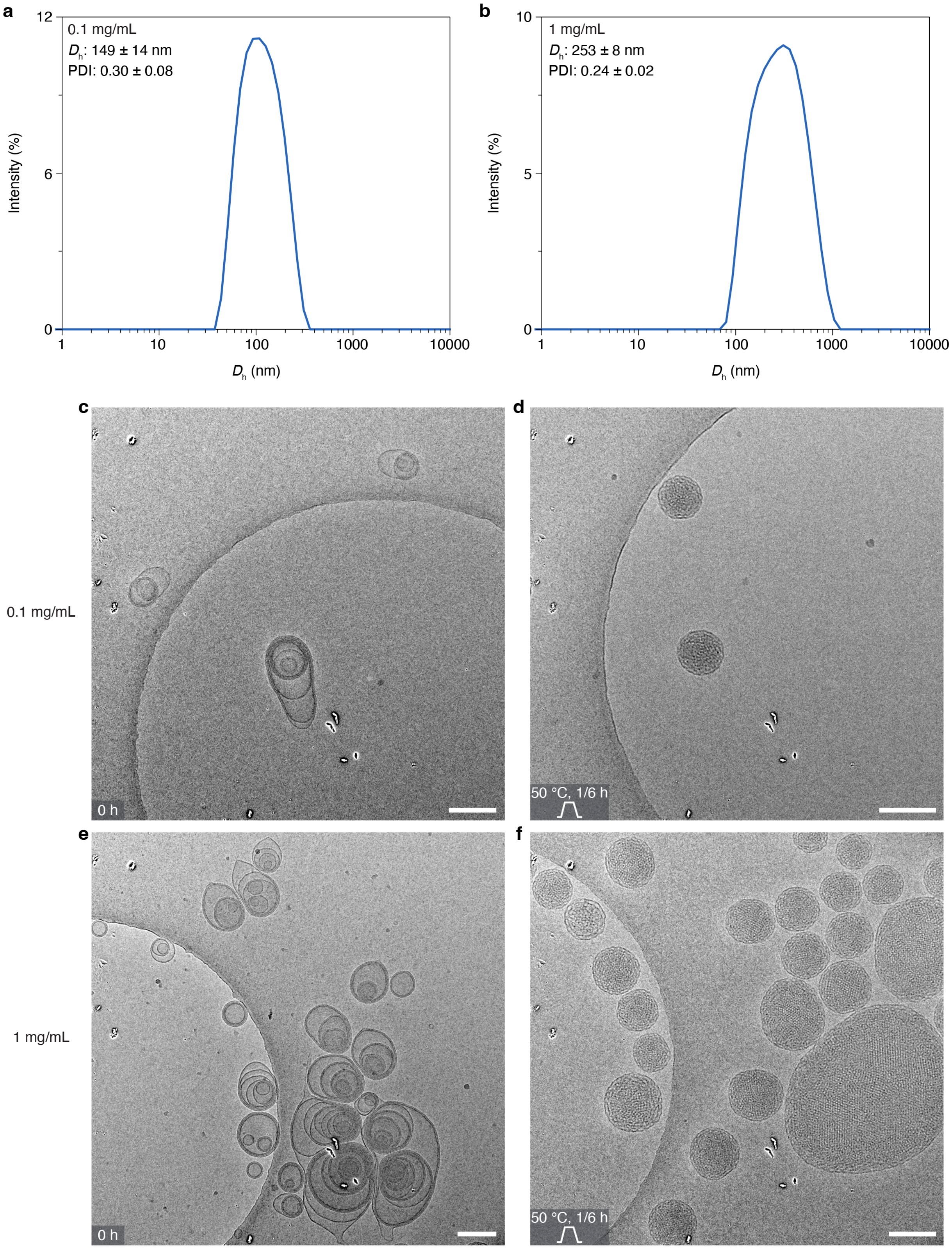

Supplementary Fig. 10 | Investigation of self-assembly concentration on morphological transitions via 50 °C annealing in the absence of ethanol. a,b D_h profiles of self-assemblies at concentrations of 0.1 mg/mL (**a**) and 1 mg/mL (**b**) determined by LS. Ethanol was removed by thorough dialysis. D_h and PDI of the self-assemblies are presented as mean ± s.d. **c,d** Cryo-

TEM images of self-assemblies of 0.1 mg/mL following ethanol removal (**c**) and assemblies annealed at 50 °C for 10 min (**d**). **e,f** Cryo-TEM images of self-assemblies of 1 mg/mL following ethanol removal (**e**) and assemblies annealed at 50 °C for 10 min (**f**). Scale bars are 200 nm.

After thorough dialysis to remove the ethanol, both samples showed a monomodal distribution with much higher PDI (0.1 mg/mL, PDI 0.30; 1 mg/mL, PDI 0.24; **Supplementary Fig. 10a,b**) than the self-assembly from concentration of 0.5 mg/mL in the manuscript (PDI 0.15). Regardless to the difference in PDI, similar nonconcentric MVVs were found to be the dominant population of both self-assemblies (**Supplementary Fig. 10c,e**). Upon annealing at 50 °C for 10 min, MVVs were replaced by inverse cubosomes (**Supplementary Fig. 10d,f**). These results confirm that the investigated concentrations of self-assemblies (0.1 – 1 mg/mL) do not pose a significant effect on the morphological transitions of self-assemblies from MVVs to cubosomes.

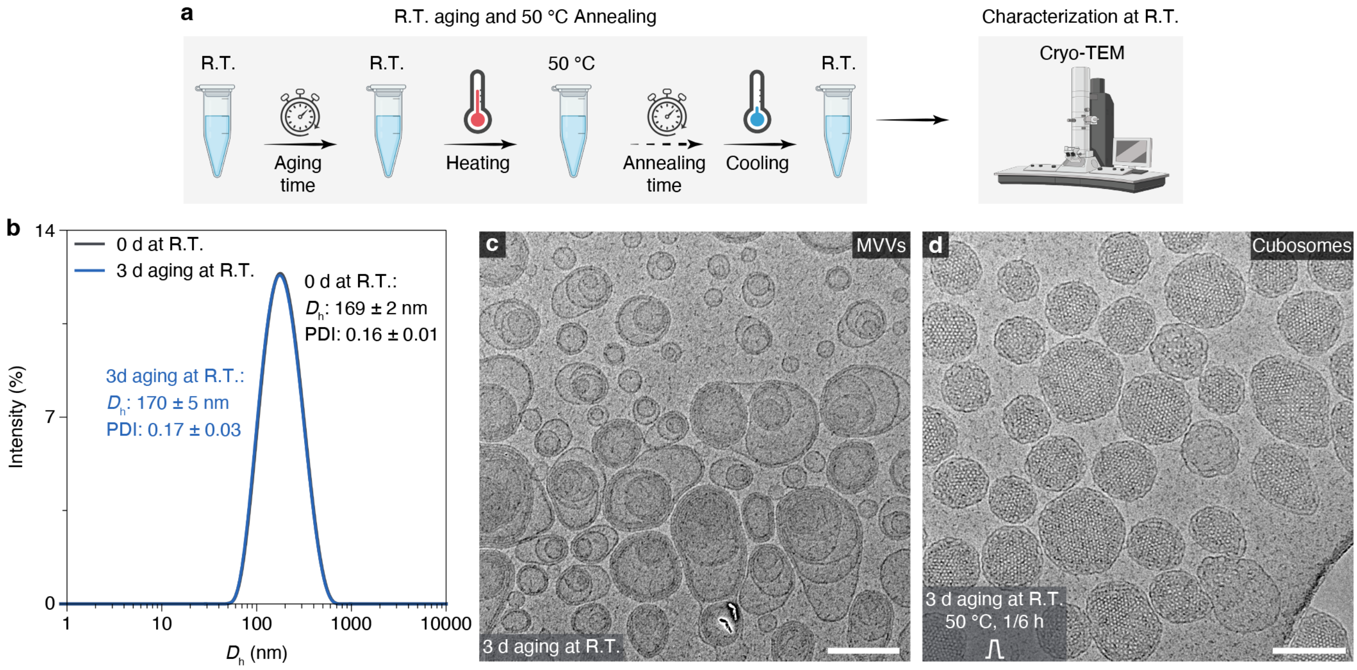

Supplementary Fig. 11 | Investigation of extended aging on the morphological transition of self-assemblies via 50 °C annealing in the absence of ethanol. a R.T. aging and the following annealing process at 50 °C and characterization of self-assemblies. Elements created in BioRender. Wilson, P. (2025) https://BioRender.com/djwnhzu. **b** D_h profiles of self-assemblies before and after 3 d aging at R.T. as determined by LS. Ethanol was removed by thorough dialysis. D_h and PDI of the self-assemblies are presented as mean ± s.d. **c,d** Cryo-TEM images of self-assemblies after 3 d aging at R.T. (**c**) and annealed at 50 °C for 10 min (**d**). Scale bars are 200 nm.

After aging the self-assemblies at R.T. for 3 d, there were no significant changes in size and PDI as measured by LS (**Supplementary Fig. 11b**). Meanwhile, the self-assemblies remained to be MVVs (**Supplementary Fig. 11c**), which is identical to the original samples (**Fig. 2d** and **Supplementary Fig. 5a**). After equilibrating at 50 °C for 10 min and cooling down to R.T., the same cubosomes were formed (**Supplementary Fig. 11d**). We can conclude that the pre-annealing aging at R.T. does not pose effects on the morphological transformation of the self-assemblies from MVVs to cubosomes.

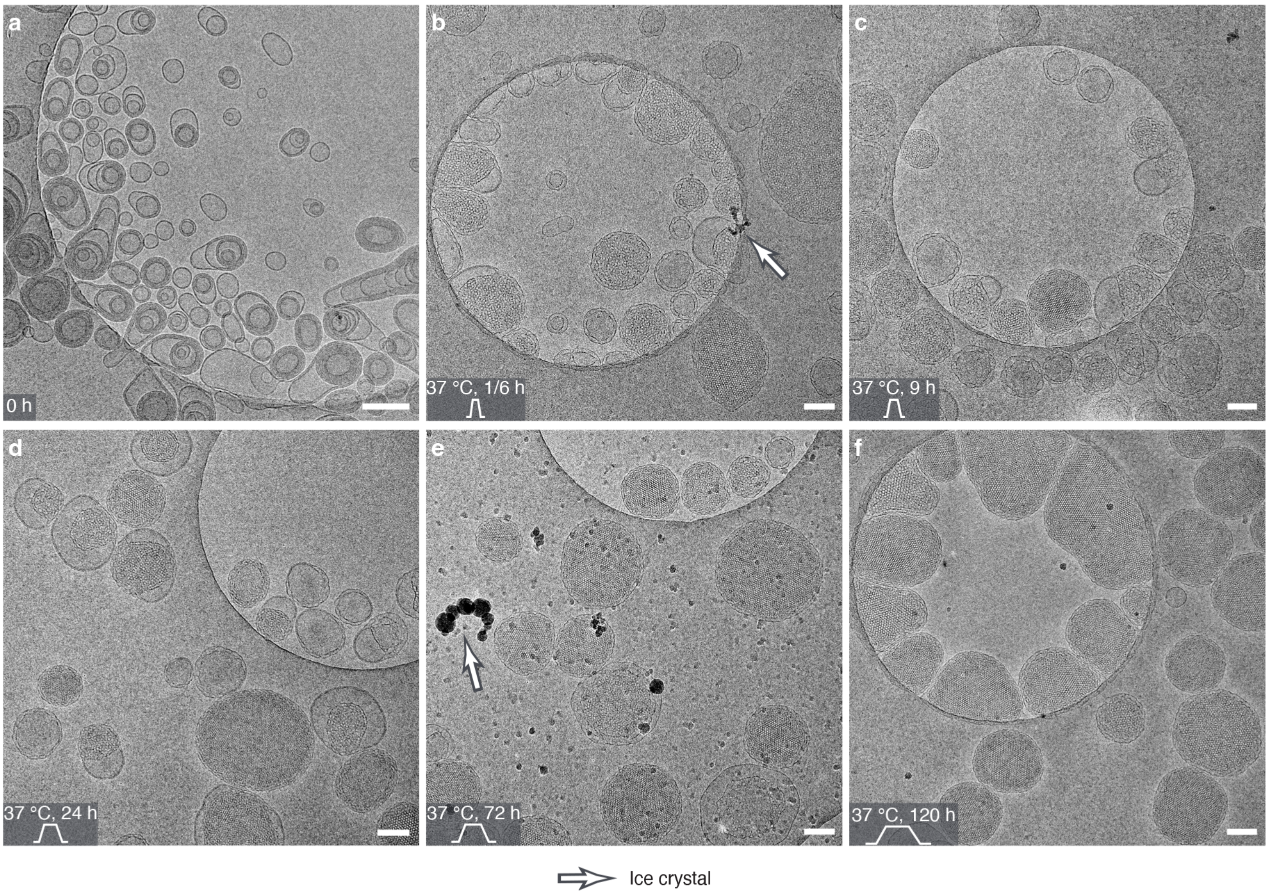

Supplementary Fig. 12 | Kinetic morphological investigation of self-assembled Janus dendrimers via 37 °C annealing in the presence of ethanol. a−f Cryo-TEM images of self-assemblies prepared by direct injection with ethanol (**a**) and assemblies annealed at 37 °C for 10 min (**b**), 9 h (**c**), 24 h (**d**), 72 h (**e**), and 120 h (**f**). Scale bars are 200 nm.

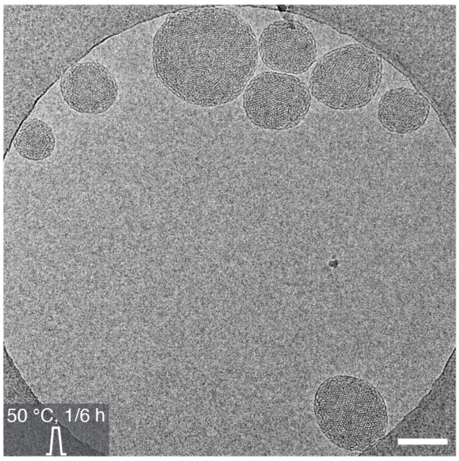

Supplementary Fig. 13 | Cryo-TEM image of self-assemblies annealed at 50 °C for 10 min in the presence of ethanol. Scale bar is 200 nm.

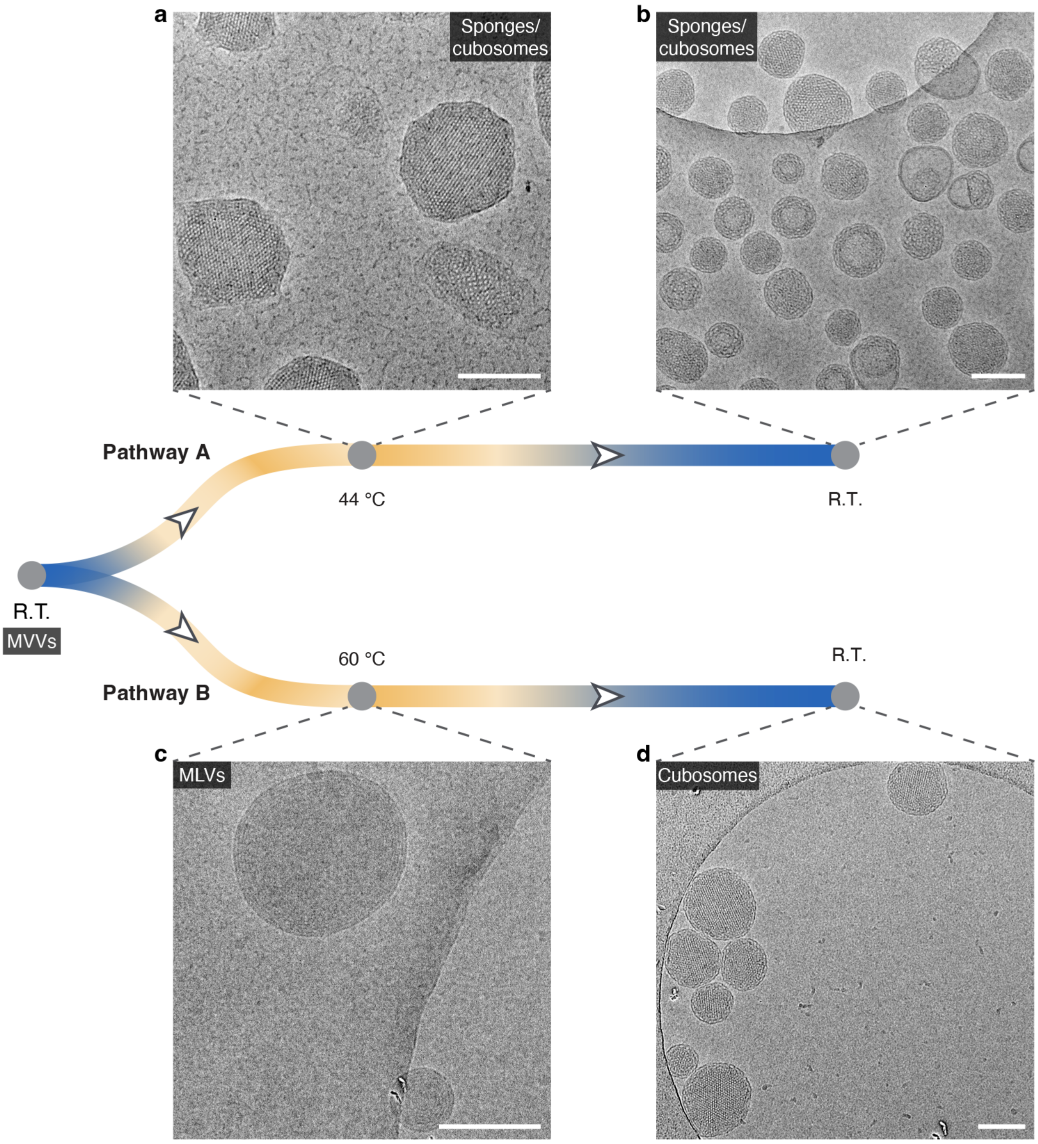

Supplementary Fig. 14 | Pathway selections of self-assemblies via 44 °C and 60 °C annealing. a–d Cryo-TEM images of self-assemblies vitrified at the indicated temperatures during the heating/cooling cycle. For samples at 44 °C and 60 °C, self-assemblies were vitrified after equilibration for 35 min and 10 min, respectively. Scale bars are 200 nm.

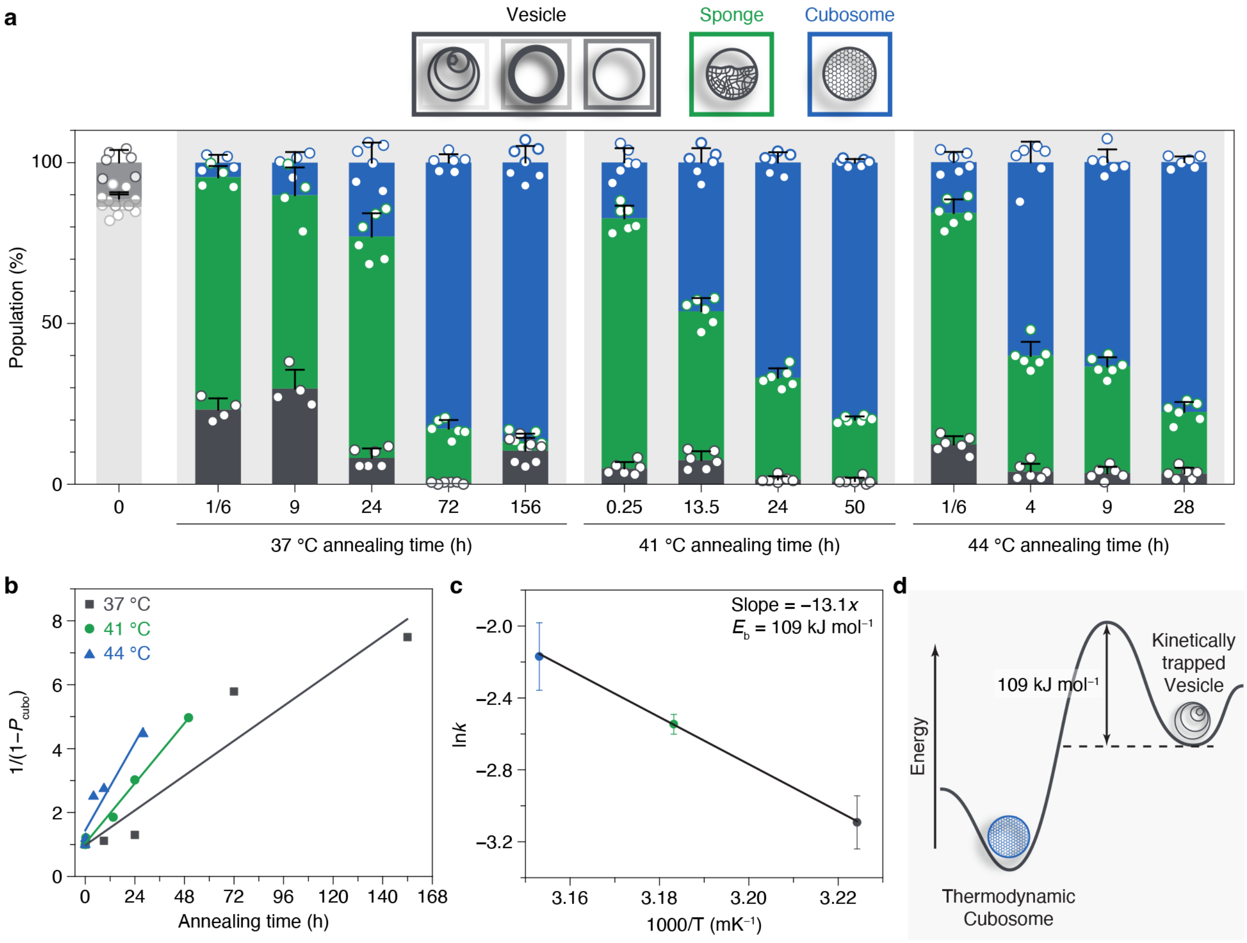

Supplementary Fig. 15 | Energy barrier of the transition from kinetically trapped vesicles to the thermodynamic cubosomes (pathway A). a Quantitative analysis of assembly populations in the absence of ethanol annealing for different time periods at different annealing temperatures. Images from multiple areas and batches were analyzed to minimize the error (Particles per condition: n > 500 for 37 °C annealing, n > 800 for 41 °C annealing, and n > 1100 for 44 °C annealing). Bar plots represent mean ± s.d. **b** Second-order kinetic plot at specific annealing temperatures against time for the transformation of MVVs to cubosomes. **c** An Arrhenius plot of the transition from MVVs to cubosomes. The k values were obtained by fitting the population of cubosomes with a second-order rate equation in (**b**). Data are presented as mean ± standard error. **d** Schematic representation of the energy landscape of self-assemblies of MVV and Cubosome separated by an energy barrier (E_b) of 109 kJ mol^{-1} (pathway **A**).

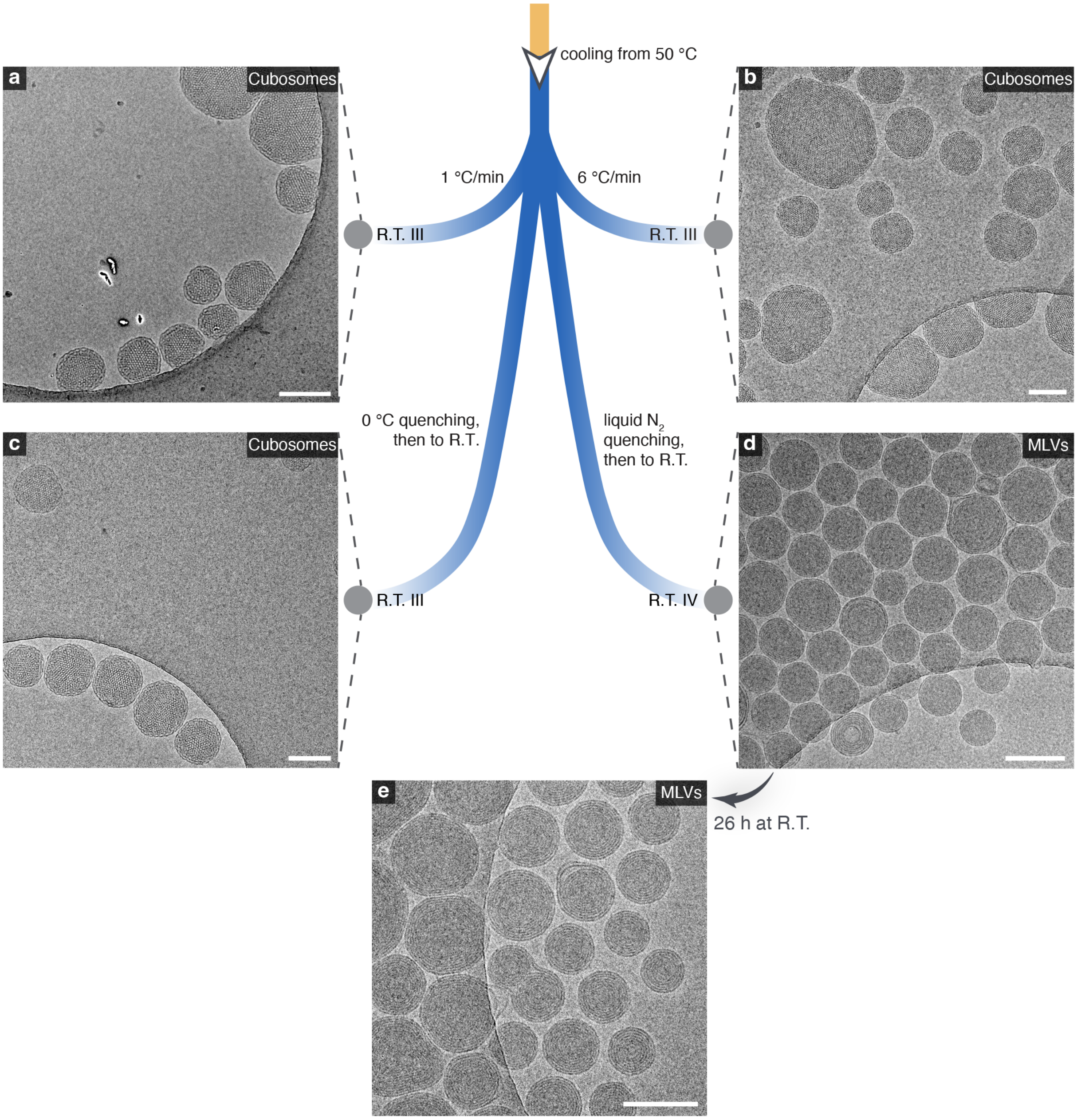

Supplementary Fig. 16 | Effect of cooling speeds from 50 °C to R.T. on the energy states of self-assemblies at R.T. a Cooling speed of 1 °C/min. **b** Cooling speed of 6 °C/min. **c** Cooling speed of 0 °C ice-water bath quenching following by equilibration at R.T. **d** Cooling speed of liquid N_2 quenching following by equilibration at R.T. **e** Stability of samples obtained in **d** after 26 h at R.T. Scale bars are 200 nm.

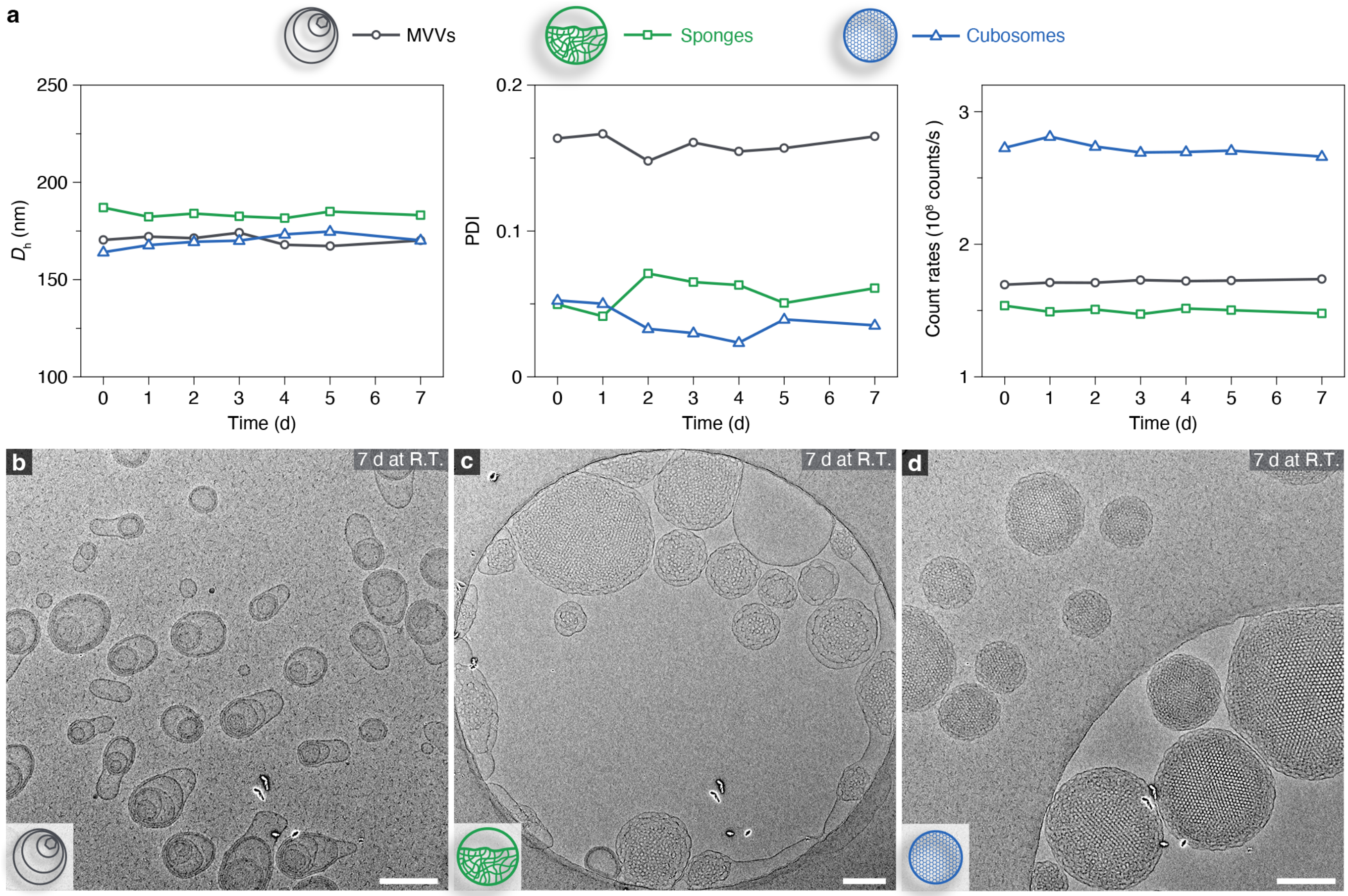

Supplementary Fig. 17 | Stability of the kinetically trapped MVVs and Sponges, and the thermodynamic cubosomes at R.T. a D_h, PDI, and count rates measured by LS of self-assembly samples over time for the kinetically trapped and thermodynamic products. **b−d** Representative cryo-TEM images of the kinetically trapped MVVs (**b**) and sponges (**c**), and the thermodynamic cubosomes (**d**) at R.T. for 7 d. Scale bars are 200 nm.

To further investigate the stability of each morphological state, each state was prepared and their stability at R.T. was tracked by LS and cryo-TEM (**Supplementary Fig. 17**). Specifically, MVVs were obtained from the original self-assembly (**Fig. 2d**), sponges were obtained from annealing at 37 °C for 10 min (**Fig. 2e**), and cubosomes were acquired from annealing at 50 °C for 10 min (**Fig. 3e**). Cryo-TEM and LS measurements did not reveal any morphological changes of each state after storing at R.T. for up to seven days, indicating that each resides in a local energy minimum.

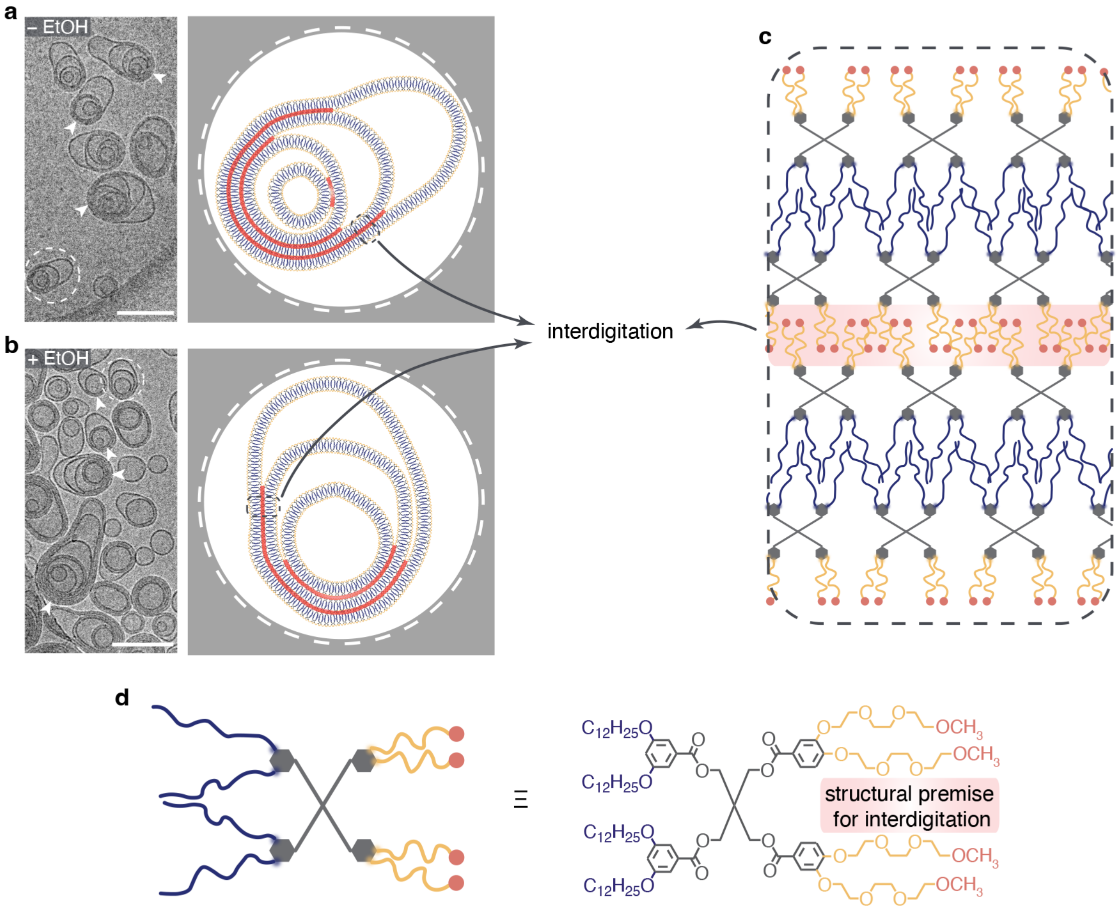

Supplementary Fig. 18 | Illustration of OEG interdigitation. Interdigitation of OEG corona of MVVs in the absence (**a**) and presence (**b**) of ethanol. Scale bars are 200 nm. **c** Schematic illustration of the interdigitation regions of OEG chains, highlighted with red-shaded areas. The attachment of the inner vesicle to the outer bilayer ("intra-attraction") results in the nonconcentric features of MVVs, as highlighted by white arrows in the corresponding cryo-TEM images. **d** Structural premise for interdigitation as depicted by the molecular structure.

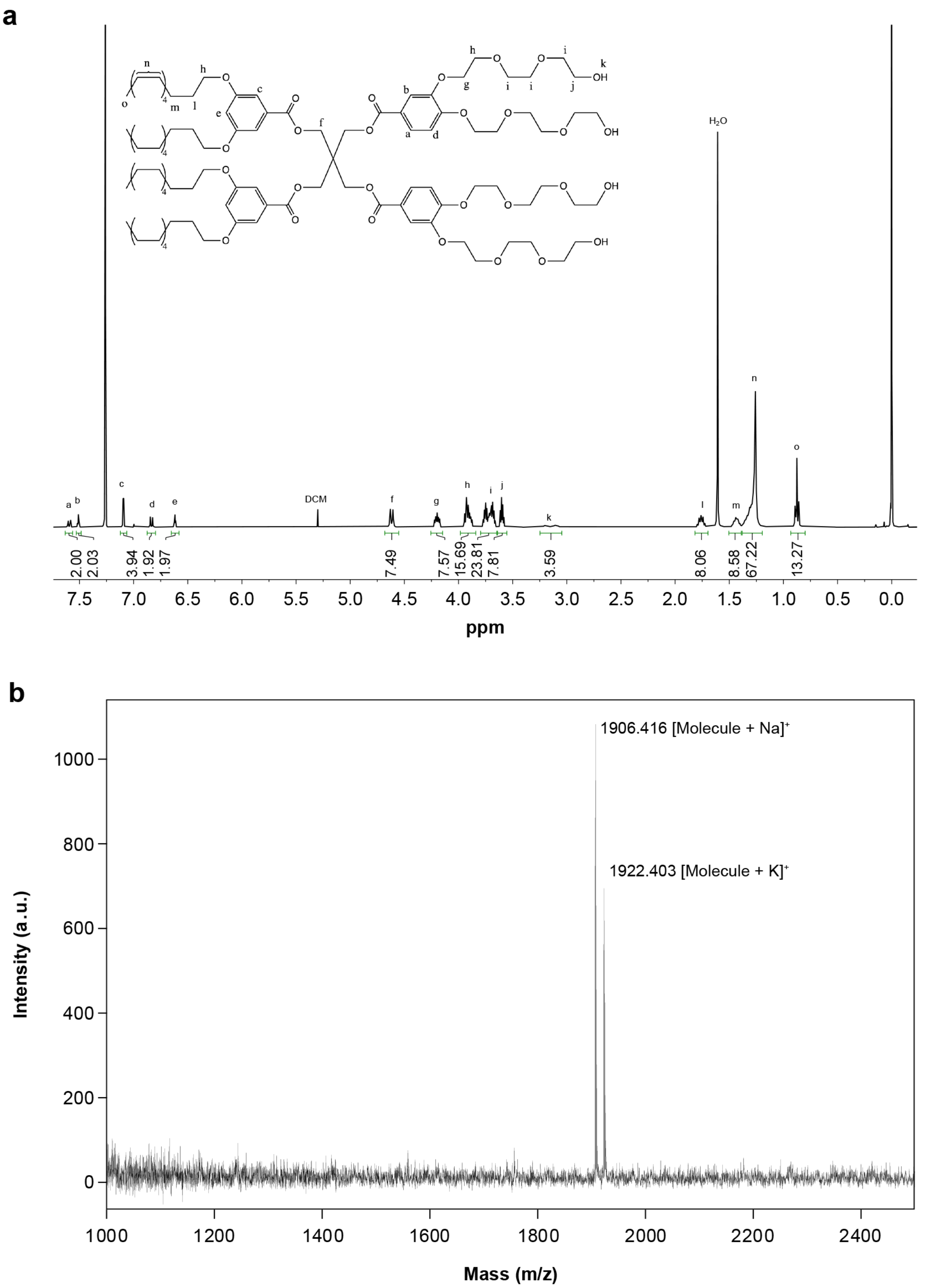

Supplementary Fig. 19 | Characterization of (3,5)12G1-PE-(3,4)-3EO-G1-(OH)$_4$ Janus dendrimer. a ^{1}H NMR in $CDCl_3$. **b** MALDI-TOF spectra.

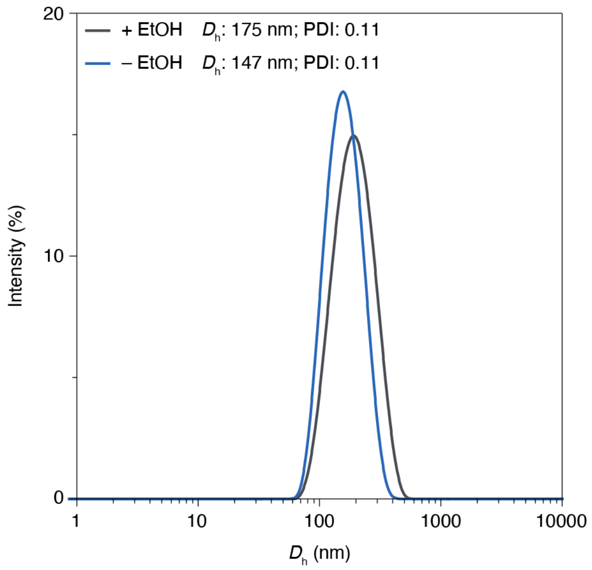

Supplementary Fig. 20 | LS characterization of self-assemblies of (3,5)12G1-PE-(3,4)-3EO-G1-(OH)$_4$ Janus dendrimers in the presence and absence of ethanol.

S4 | References

(1) Percec, V.; Wilson, D. A.; Leowanawat, P.; Wilson, C. J.; Hughes, A. D.; Kaucher, M. S.; Hammer, D. A.; Levine, D. H.; Kim, A. J.; Bates, F. S.; Davis, K. P.; Lodge, T. P.; Klein, M. L.; DeVane, R. H.; Aqad, E.; Rosen, B. M.; Argintaru, A. O.; Sienkowska, M. J.; Rissanen, K.; Nummelin, S.; Ropponen, J. Self-assembly of Janus dendrimers into uniform dendrimersomes and other complex architectures. *Science* **2010**, *328*, 1009–1014.

(2) Berghausen, J.; Zipfel, J.; Lindner, P.; Richtering, W. Influence of Water-Soluble Polymers on the Shear-Induced Structure Formation in Lyotropic Lamellar Phases. *J. Phys. Chem. B* **2001**, *105*, 11081–11088.

(3) Potter, M.; Najer, A.; Klockner, A.; Zhang, S.; Holme, M. N.; Nele, V.; Che, J.; Massi, L.; Penders, J.; Saunders, C.; Doutch, J. J.; Edwards, A. M.; Ces, O.; Stevens, M. M. Controlled dendrimersome nanoreactor system for localized hypochlorite-induced killing of bacteria. *ACS Nano* **2020**, *14*, 17333−17353.

(4) Zhang, D.; Atochina-Vasserman, E. N.; Maurya, D. S.; Liu, M.; Xiao, Q.; Lu, J.; Lauri, G.; Ona, N.; Reagan, E. K.; Ni, H.; Weissman, D.; Percec, V. Targeted delivery of mRNA with one-component ionizable amphiphilic Janus dendrimers. *J. Am. Chem. Soc.* **2021**, *143*, 17975–17982.

(5) Zhang, D.; Atochina-Vasserman, E. N.; Maurya, D. S.; Huang, N.; Xiao, Q.; Ona, N.; Liu, M.; Shahnawaz, H.; Ni, H.; Kim, K.; Billingsley, M. M.; Pochan, D. J.; Mitchell, M. J.; Weissman, D.; Percec, V. One-Component Multifunctional Sequence-Defined Ionizable Amphiphilic Janus Dendrimer Delivery Systems for mRNA. *J. Am. Chem. Soc.* **2021**, *143*, 12315−12327.

(6) Zhang, D.; Atochina-Vasserman, E. N.; Lu, J.; Maurya, D. S.; Xiao, Q.; Liu, M.; Adamson, J.; Ona, N.; Reagan, E. K.; Ni, H.; Weissman, D.; Percec, V. The Unexpected Importance of the Primary Structure of the Hydrophobic Part of One-Component Ionizable Amphiphilic Janus Dendrimers in Targeted mRNA Delivery Activity. *J. Am. Chem. Soc.* **2022**, *144*, 4746−4753.

(7) Hamley, I. W. Diffuse scattering from lamellar structures. *Soft Matter* **2022**, *18*, 711–721.

(8) Frielinghaus, H. Small-angle scattering model for multilamellar vesicles. *Phys. Rev. E: Stat., Nonlinear. Biol., Soft Matter Phys.* **2007**, *76*, 051603.

(9) Seneviratne, R.; Coates, G.; Xu, Z.; Cornell, C. E.; Thompson, R. F.; Sadeghpour, A.;

Maskell, D. P.; Jeuken, L. J. C.; Rappolt, M.; Beales, P. A. High Resolution Membrane Structures within Hybrid Lipid-Polymer Vesicles Revealed by Combining X-Ray Scattering and Electron Microscopy. *Small* **2023**, *19*, e2206267.

(10) Wang, J. Experimental charge density from electron microscopic maps. *Protein Sci.* **2017**, *26*, 1619–1626.

(11) Sharma, K. D.; Heberle, F. A.; Waxham, M. N. Visualizing lipid membrane structure with cryo-EM: past, present, and future. *Emerg. Top. Life Sci.* **2023**, *7*, 55–65.

(12) Angelov, B.; Angelova, A.; Mutafchieva, R.; Lesieur, S.; Vainio, U.; Garamus, V. M.; Jensen, G. V.; Pedersen, J. S. SAXS investigation of a cubic to a sponge (L3) phase transition in self-assembled lipid nanocarriers. *Phys. Chem. Chem. Phys.* **2011**, *13*, 3073–3081.

(13) Caselli, L.; Conti, L.; De Santis, I.; Berti, D. Small-angle X-ray and neutron scattering applied to lipid-based nanoparticles: Recent advancements across different length scales. *Adv. Colloid Interface Sci.* **2024**, *327*, 103156.

(14) Sagalowicz, L.; Acquistapace, S.; Watzke, H. J.; Michel, M. Study of Liquid Crystal Space Groups Using Controlled Tilting with Cryogenic Transmission Electron Microscopy. *Langmuir* **2007**, *23*, 12003–12009.
